# Photodynamic Therapy for Juxtapapillary Retinal Capillary Hemangioma

**DOI:** 10.1155/2014/756840

**Published:** 2014-03-03

**Authors:** Panagiotis G. Mitropoulos, Irini P. Chatziralli, Vasileios G. Peponis, Vasileia A. Tsiotra, Efstratios A. Parikakis

**Affiliations:** 2nd Eye Clinic, “Ophthlmiatrion Athinon,” 26 Eleftheriou Venizelou Street, 10672 Athens, Greece

## Abstract

Various treatment modalities have been described for retinal capillary hemangioma. Our purpose is to present a case of juxtapapillary retinal capillary hemangioma treated with photodynamic therapy. A 69-year-old woman with no previous ocular history presented with blurred vision and photopsias in the right eye three months ago. At presentation, her best corrected visual acuity was 6/9 in the right eye and 6/6 in the left eye. The anterior segment was totally normal and IOP was normal in both eyes as well. Dilated fundoscopy revealed a yellowish, well-circumscribed, elevated area with blood vessels, on the inferior margin of the right optic disc, as optic disc edema. Fluorescein angiography and angiogram with indocyanine green confirmed the diagnosis of juxtapapillary retinal capillary hemangioma. The patient was treated with photodynamic therapy with verteporfin and three months later her visual acuity was 6/7.5 in the right eye, while the lesion was slightly smaller. These findings remained stable at the one-year follow-up. In conclusion, photodynamic therapy offers promising anatomical and functional results for juxtapapillary retinal capillary hemangioma, providing visual acuity improvement or even stabilization and restriction of enlargement of the lesion.

## 1. Introduction

Retinal capillary hemangioma (RCH) or hemangioblastoma is an uncommon benign vascular tumor and may occur sporadically (54%) or as a manifestation of von Hippel-Lindau (VHL) disease (46%) [1–4]. Although it is usually considered as a solitary unilateral tumor, when associated with VHL disease, up to 50% of the cases present multifocal or bilateral involvement [1–8]. The clinical course of the disease is usually progressive and difficult to predict [7, 8]. As the RCH enlarges, it can cause complications, such as exudation, subretinal fluid accumulation, macular edema, and exudative retinal detachment, resulting in visual deterioration. Furthermore, glial proliferation can lead to epiretinal membrane development or tractional retinal detachment [1–8]. RCH can be located at the disc (papillary), juxtapapillary, or at the peripheral retina, occurring most commonly at the temporal side of the disc [1–8].

Treatment depends on the location and size of the RCH and varies from observation to radiotherapy, cryotherapy, transpupillary thermotherapy, laser photocoagulation, photodynamic treatment (PDT), antivascular endothelial growth factor (anti-VEGF) agents, intravitreal triamcinolone, vitreoretinal surgery, or combination of treatment modalities [5, 6, 9–28]. There are few case reports and case series, using PDT for juxtapapillary RCH, having mixed anatomical and functional results [5, 6, 17–19, 21, 24, 27]. Herein, we present the case of a woman with juxtapapillary RCH treated with PDT, exhibiting improvement in the visual acuity and stabilization of the disease.

## 2. Case Presentation

**Figure 1:**
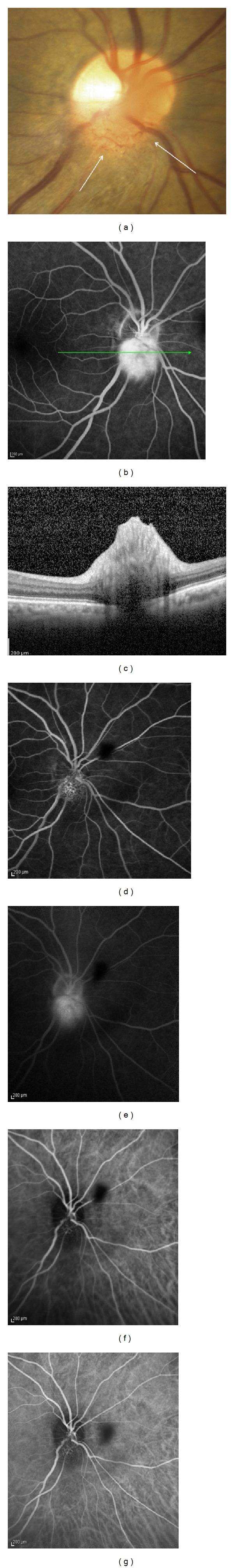
(a) Through. (c) Fundus photo, fluorescein angiography late phase, and optical coherence tomography at presentation. (d)-(e) Fluorescein angiography early and late phase at presentation. (f)-(g) Indocyanine green angiography early and late phase at presentation.

A 69-year-old woman, with no previous ocular history, presented with blurred vision and photopsias in the right eye three months ago. At presentation, her best-corrected visual acuity (BCVA) was 6/9 in the right eye and 6/6 in the left eye. Dilated fundoscopy revealed a yellowish, elevated, well-circumscribed lesion with blood vessels, of about one optic disc diameter on the inferior margin of the right optic disc. There were neither peripheral lesions identified nor other ocular abnormalities. Fluorescein angiography (FA) demonstrated early hyperfluorescence of the tumor vessels and some progressive leakage in the late phase of the angiogram (Figure 1), confirming the diagnosis of juxtapapillary RCH. Indocyanine green angiography (ICGA) and optic coherence tomography (OCT) reinforced the diagnosis (Figure 1). Systemic investigations showed no evidence of VHL disease.

After explaining to the patient the various treatment modalities against RCH and potential complications, a decision was made to treat the lesion with PDT using Verteporfin (Visudyne, Novartis Pharmaceuticals, Basel, Switzerland). Written informed consent was obtained from the patient. Standard PDT was performed using Visudyne infusion at a dose of 6 mg/m^2^ body surface area for 10 min. Five minutes after completion of the infusion, light exposure was performed with a diode laser (689 nm) at an intention of 600 mW/cm^2^ for 83 seconds. The spot size was adjusted to cover the largest diameter of the hemangioma without extension into surrounding retina. A single exposure was applied in one treatment session.

**Figure 2:**
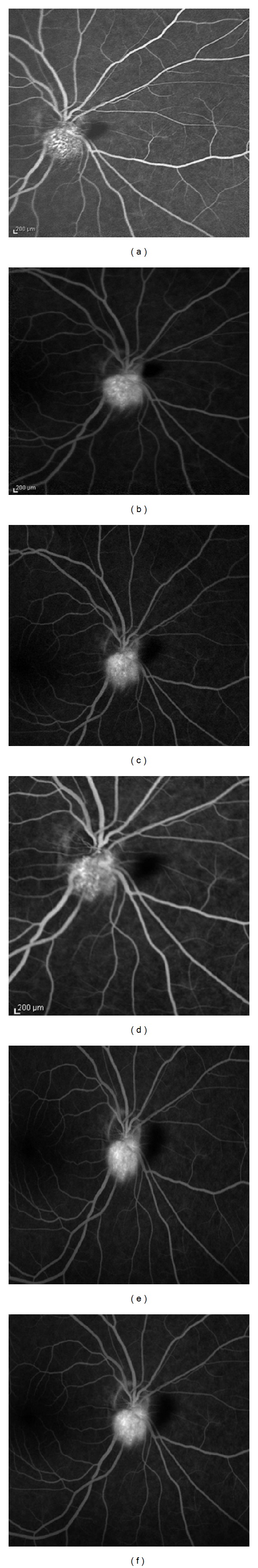
(a) Through. (c) Fluorescein angiography early and late phase three months after photodynamic therapy. (d) Though. (f) Fluorescein angiography early and late phase one year after photodynamic therapy, showing stability of the lesion.

Follow-up visits, including BCVA measurement, fundoscopy, OCT, and FA, were scheduled one month and three months following PDT session and continued up to 12 months. Three months following treatment, BCVA improved to 6/7.5 with no adverse effects from the treatment. There was a slight regression of the tumor, as it is depicted on FA (Figure 2). At the end of the one-year follow-up, her BCVA remained stable at 6/7.5, the size of the lesion was slightly smaller compared to baseline, and neither subretinal fluid nor recurrence was observed.

## 3. Discussion

The treatment of juxtapapillary RCH remains challenging due to the vicinity to the optic nerve and is based on size and location of the lesion, as well as the presence of associated complications [9]. By and large, juxtapapillary RCH is treated if visual acuity deteriorates and the treatment goal is preservation of visual acuity and visual field, without destruction of the function of the retina around the tumor [9].

Careful observation is recommended if the RCH is very small (up to 500 *μ*m), is not associated with exudation or subretinal fluid, and is not vision threatening, as juxtapapillary RCH may remain stable for longer periods of time compared to extrapapillary lesions [9]. Interestingly, spontaneous regression of RCH has been reported [10, 11]. Transpupillary thermoplasty has been used in the treatment of two reported cases of juxtapapillary RCH, which resulted in complete fibrosis and atrophy [1, 9]. Laser photocoagulation is used to treat small RCH (up to 1.5 mm) in the posterior retina in eyes with clear media but carries additional risk for juxtapapillary RCH due to the proximity to the optic nerve, as it usually requires multiple and intense burns and damages the nerve fiber layer, causing usually an arcuate scotoma [5, 6, 9]. Cryotherapy is preferable to photocoagulation for larger RCH (more than 3 mm in diameter), which is located anteriorly with a significant amount of subretinal fluid [5, 6, 9].

Moreover, it has been found that patients with RCH have elevated ocular levels of VEGF, suggesting that RCH may depend on VEGF. Currently, in the era of anti-VEGF agents, there are several reports using anti-VEGF injections for the treatment of RCH, presenting variable results. Ach et al. used intravitreal bevacizumab for midperipheral RCH and reported that repeated intravitreal injections have inhibited the growth of the lesion [14], in line with Dahr et al. who presented two cases of large peripheral angiomas treated with intravitreal pegaptanib, having improvement in visual acuity but no change in fluorescein angiography at the one-year follow-up [15]. Accordingly, Clelala et al. reported a case of juxtapapillary RCH which was treated with a single injection of ranibizumab, demonstrating mild shrinkage and reduced vascularization at the six-month follow-up, although visual acuity and visual field remained unchanged [16].

Furthermore, combined therapy with anti-VEGF agents and PDT has shown promising results against the treatment of RCH. Mennel et al. reported a case of RCH in the right eye treated with PDT and five injections of bevacizumab, showing improvement in visual acuity and stabilization of the lesion [18]. Ziemssen et al. treated a juxtapapillary RCH with a single intravitreal injection of bevacizumab and PDT and found regression of the hemangioma and increase in visual acuity at the one-year follow-up [19]. Additionally, Fong et al. used intravitreal ranibizumab and PDT combined with pars plana vitrectomy for the treatment of a juxtapapillary RCH, which was associated with macular edema and epiretinal membrane, and reported improvement in visual acuity that remained stable at the one-year follow-up [17].

Photodynamic therapy using Visudyne is a promising treatment alternative. PDT is a nonthermal, photobiochemical procedure, offering site-specific vascular occlusion and tumor destruction, causing minimal damage to the adjacent neural structures [24]. The safety and efficacy of PDT in choroidal neovascularization of several causes, such as age-related macular degeneration or polypoidal choroidal vasculopathy, are well established [29]. It has been also found to treat choroidal hemangioma and other retinal tumors with consequent regression of the lesion and resolution of subretinal fluid [26, 30]. Furthermore, the vascular nature of RCH allows pooling of verteporfin, enabling a site-specific target for treatment [26].

As choroidal and retinal hemangioma share a common histopathology in compromised angiomatous vessels, there are various reports suggesting that PDT may have encouraging results in RCH treatment [6, 18, 19, 22–24]. Nevertheless, Schmidt-Erfurth et al. reported no visual acuity improvement in five eyes with RCH treated with PDT using Visudyne, although there was tumor regression, as well as resolution of macular exudates after treatment, without affecting the adjacent neural structures [26]. The authors attributed the discrepancy between anatomical and functional results to vascular complications, such as optic nerve ischemia and retinal vessel occlusions [26]. Golshevsky and O'Day found subjective improvement in visual acuity and stabilization of the lesion in a juxtapapillary RCH treated with PDT and proposed PDT as a useful treatment for juxtapapillary RCH [6], in line with other authors [18, 19, 22–24]. In our case, the patient received a single session of PDT and presented improvement in visual acuity, as well as slight shrinkage of the lesion.

The differential diagnosis of a juxtapapillary RCH can be difficult, as it may be easily misdiagnosed as papilloedema, papillitis, granulomatous diseases, or pepipapillary subretinal neovascularization. Therefore, fluorescein angiography is of great importance for accurate diagnosis. Additionally, in cases of RCH, it is essential to rule out the possibility of VHL disease, examining carefully the patient for presence of additional angiomas in the peripheral retina. Furthermore, systemic evaluation should be performed to exclude the presence of tumors in the brain, spinal cord, kidney, and adrenal glands [5, 28]. B-scan ultrasonography may also be of great benefit, so as to rule out choroidal melanoma and osteoma [5, 6, 28].

Taking as a whole, herein we report a case of juxtapapillary RCH treated with a single session of PDT with Visudyne, presenting improvement in visual acuity from 6/9 to 6/7.5 and slight shrinkage of the lesion size. The patient's visual acuity, as well as fluorescein angiography image, remained stable at the one-year follow-up, suggesting that PDT may be a treatment alternative with promising results against juxtapapillary RCH. Further studies with large number of patients are needed to reach a safe conclusion.

## References

[1] Singh AD, Nouri M, Shields CL, Shields JA, Smith AF (2001). Retinal capillary hemangioma: a comparison of sporadic cases and cases associated with von Hippel-Lindau disease. *Ophthalmology*.

[2] Magee MA, Kroll AJ, Lou PL, Ryan EA (2006). Retinal capillary hemangiomas and von Hippel-Lindau disease. *Seminars in Ophthalmology*.

[3] Schindler RF, Sarin LK, MacDonald PR (1975). Hemangiomas of the optic disc. *Canadian Journal of Ophthalmology*.

[4] Gass JD, Braunstein R (1980). Sessile and exophytic capillary angiomas of the juxtapapillary retina and optic nerve head. *Archives of Ophthalmology*.

[5] Sachdeva R, Dadgostar H, Kaiser PK, Sears JE, Singh AD (2010). Verteporfin photodynamic therapy of six eyes with retinal capillary haemangioma. *Acta Ophthalmologica*.

[6] Golshevsky JR, O’Day J (2005). Photodynamic therapy in the management of juxtapapillary capillary haemangiomas. *Clinical and Experimental Ophthalmology*.

[7] McCabe CM, Flynn HW, Shields CL (2000). Juxtapapillary capillary hemangiomas: clinical features and visual acuity outcomes. *Ophthalmology*.

[8] Kuo M-T, Kou H-K, Kao M-L, Tsai M-H, Chen Y-J, Lin S-A (2002). Retinal capillary hemangiomas: clinical manifestations and visual prognosis. *Chang Gung Medical Journal*.

[9] McDonald HR (2003). Diagnostic and therapeutic challenges. Juxtapapillary retina capillary hemangioma. *Retina*.

[10] Milewski SA (2002). Spontaneous regression of a capillary hemangioma of the optic disc. *Archives of Ophthalmology*.

[11] Molina MC, Paulo JD, Donado JH (2008). Spontaneous regression of a retinal capillary hemangioma. *Retina*.

[12] Russo V, Stella A, Barone A, Scott IU, Noci ND (2012). Ruthenium-106 brachytherapy and intravitreal bevacizumab for retinal capillary hemangioma. *International Ophthalmology*.

[13] Cheung J, Lee GK, Tham CC (2012). A large retinal capillary hemangioma in the anterior retina treated with photodynamic therapy. *Case Reports in Ophthalmology*.

[14] Ach T, Thiemeyer D, Hoeh AE, Schaal KB, Dithmar S (2010). Intravitreal bevacizumab for retinal capillary haemangioma: longterm results. *Acta Ophthalmologica*.

[15] Dahr SS, Cusick M, Rodriguez-Coleman H (2007). Intravitreal anti-vascular endothelial growth factor therapy with pegaptanib for advanced von Hippel-Lindau disease of the retina. *Retina*.

[16] Chelala E, Dirani A, Fadlallah A (2013). Intravitreal anti-VEGF injection for the treatment of progressive juxtapapillary retinal capillary hemangioma: a case report and mini review of the literature. *Journal of Clinical Ophthalmology*.

[17] Fong AHC, Li KKW, Wong D (2011). Intravitreal ranibizumab, photodynamic therapy, and vitreous surgery for the treatment of juxtapapillary retinal capillary hemangioma. *Graefe’s Archive for Clinical and Experimental Ophthalmology*.

[18] Mennel S, Meyer CH, Callizo J (2010). Combined intravitreal anti-vascular endothelial growth factor (Avastin) and photodynamic therapy to treat retinal juxtapapillary capillary haemangioma. *Acta Ophthalmologica*.

[19] Ziemssen F, Voelker M, Inhoffen W, Bartz-Schmidt KU, Gelisken F (2007). Combined treatment of a juxtapapillary retinal capillary haemangioma with intravitreal bevacizumab and photodynamic therapy. *Eye*.

[20] Wittenberg L, Ma P (2008). Treatment of a von Hippel-Lindau retinal capillary hemangioma with photodynamic therapy. *Canadian Journal of Ophthalmology*.

[21] Papastefanou VP, Pilli S, Stinghe A, Lotery AJ, Cohen VM (2013). Photodynamic therapy for retinal capillary hemangioma. *Eye*.

[22] Bakri SJ, Sears JE, Singh AD (2005). Transient closure of a retinal capillary hemangioma with verteporfin photodynamic therapy. *Retina*.

[23] Aaberg TM, Aaberg TM, Martin DF, Gilman JP, Myles R (2005). Three cases of large retinal capillary hemangiomas treated with verteporfin and photodynamic therapy. *Archives of Ophthalmology*.

[24] Atebara NH (2002). Retinal capillary hemangioma treated with verteporfin photodynamic therapy. *The American Journal of Ophthalmology*.

[25] Toyokawa N, Kimura H, Kuroda S (2010). Juxtapapillary capillary hemangioma treated by intravitreal injection of bevacizumab combined with posterior subtenon injection of triamcinolone acetonide. *Japanese Journal of Ophthalmology*.

[26] Schmidt-Erfurth UM, Kusserow C, Barbazetto IA, Laqua H (2002). Benefits and complications of photodynamic therapy of papillary capillary hemangiomas. *Ophthalmology*.

[27] Baba T, Kitahashi M, Kubota-Taniai M, Oshitari T, Yamamoto S (2011). Subretinal hemorrhage after photodynamic therapy for juxtapapillary retinal capillary hemangioma. *Case Reports in Ophthalmology*.

[28] Singh AD, Nouri M, Shields CL, Shields JA, Perez N (2002). Treatment of retinal capillary hemangioma. *Ophthalmology*.

[29] Kaiser PK (2006). Treatment of age-related macular degeneration with photodynamic therapy (TAP) study group. Verteporfin therapy of subfoveal choroidal neovascularization in age-related macular degeneration: 5-year results of two randomized clinical trials with an open-label extension. TAP report no. 8. *Graefe’s Archive for Clinical and Experimental Ophthalmology*.

[30] Barbazetto I, Schmidt-Erfurth U (2000). Photodynamic therapy of choroidal hemangioma: two case reports. *Graefe’s Archive for Clinical and Experimental Ophthalmology*.

